# Case Report: Severe lead poisoning due to exposure to ayurvedic herbal medicine

**DOI:** 10.3389/fped.2025.1692561

**Published:** 2025-10-30

**Authors:** Gabriella Cericola, Alexander Puzik, Sarah Salou, Ayami Yoshimi-Nöllke, Charlotte Niemeyer, Tobias Feuchtinger, Fred Henretig, Luke Yip, Siegfried Krell, Simone Hettmer

**Affiliations:** ^1^ Division of Pediatric Hematology and Oncology, Faculty of Medicine, Children’s Hospital, University Medical Center of Freiburg, University of Freiburg, Freiburg, Germany; ^2^ Department of Pediatrics, Perelman School of Medicine, University of Pennsylvania, Philadelphia, PA, United States; ^3^ National Center for Environmental Health, Centers for Disease Control and Prevention, Atlanta, GA, United States; ^4^ Medical Laboratory Bremen, MVZ, Bremen, Germany; ^5^ University Medicine Halle, Pediatrics 1, Martin Luther University Halle, Halle (Saale), Germany

**Keywords:** lead poisoning, ayurvedic medicine, heavy metal toxicity, chelation therapy, pediatric environmental health, complementary and alternative medicine

## Abstract

**Introduction:**

Lead is an environmental toxin that may cause severe damage to vital organs including the brain, kidneys, liver, and bones. Children are particularly susceptible due to higher rates of gastrointestinal absorption and detrimental effects of lead on their developing nervous systems.

**Methods:**

This report highlights the case of a 4-year-old boy with severe lead poisoning resulting from prolonged exposure to ayurvedic herbal supplements.

**Results:**

The child was initially admitted with anemia, arterial hypertension, abdominal pain, and mild neurological impairment. His blood smear revealed pronounced basophilic stippling of erythrocytes. His venous blood lead level (BLL) was markedly elevated at 123 μg/dl. Detailed review of his history uncovered that he had been ingesting an ayurvedic herbal medicine for asthma. He received chelation therapy with 2,3-dimercapto-1-propanesulfonic acid and calcium disodium EDTA, resulting in rapid resolution of symptoms and gradually decreasing BLLs.

**Conclusions:**

The case is a striking example of the significant health risks due to heavy metal contamination in ayurveda products. Better strategies to control the composition of ayurvedic products and educate families about their possible heavy metal contamination are essential to reduce the risk of lead poisoning.

## Introduction

In Ayurveda, a traditional system of medicine from India, health is viewed as balance of body, mind, and spirit. Ayurveda treatments involve individualized combinations of diet, lifestyle practices (often including massages, yoga and meditation) and—in certain traditions (e.g., Rasa Shastra) intake of mineral/ metal preparations (1). A 2020 United Nations Children's Fund (UNICEF) study revealed that one third of the world's child population have blood lead levels (BLLs) above 3.5 µg/dl (2) [current U.S. CDC blood lead reference level (3)]. Lead exposure was responsible for nearly half of the 2 million deaths linked to chemical exposures in 2019, 30% of cases of idiopathic intellectual disability, 4.6% of cardiovascular disease, and 3% of chronic kidney disease worldwide (4). This report presents the case of a 4-year-old boy with symptomatic lead poisoning resulting from prolonged exposure to Ayurvedic supplements and highlights the serious risks posed by lead-containing Ayurvedic products.

## Case report

**Figure 1:**
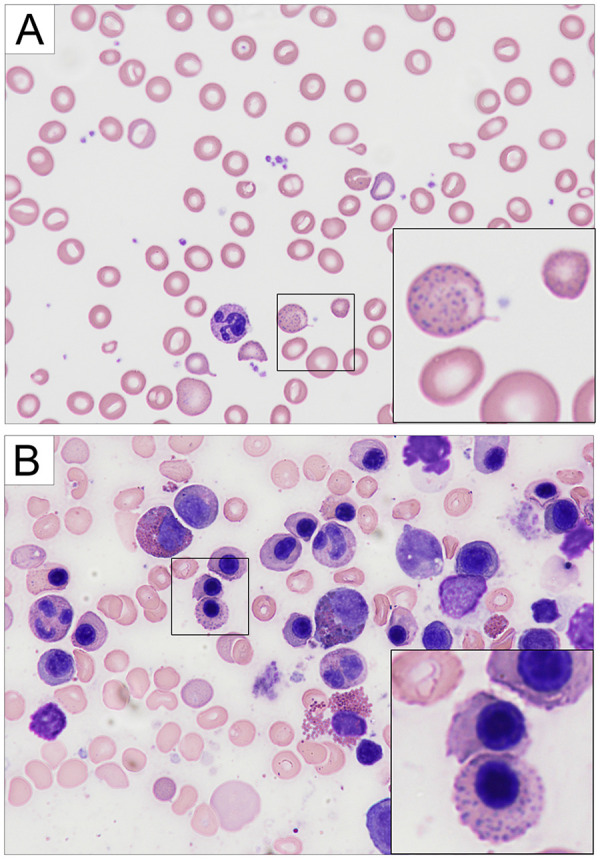
Hematology findings. The patient presented with normochromic anemia. **(A)** Giemsa-stained peripheral blood showed marked basophilic stippling of erythrocytes. **(B)** The bone marrow was normocellular with adequate representation of megakaryocytes. Both images are shown at 600×.

A 4-year-old boy of Italian heritage on the mother's side and German heritage on the father's side of the family was admitted to an academic medical center in Germany with fatigue, weakness, and normochromic anaemia. His blood pressure was 101/53 mmHg, and his heart rate heart rate was 110/min. Physical examination was age-appropriate except for pale skin. His hemoglobin level was critically low at 4.7 g/dl with an elevated reticulocyte count (252%), no signs of hemolysis, normal white blood cell and platelet counts. Bone marrow biopsy revealed a normocellular marrow with adequate representation of megakaryocytes, unremarkable myelopoiesis and mild hemophagocytosis. Erythropoiesis was hyperactive, left-shifted and mildly dysplastic with basophilic stippling (Figure 1B). Hemoglobin levels spontaneously recovered to 6.4 g/dl, and he was discharged. The patient was readmitted six days later with worsening abdominal discomfort, behavioural changes and arterial hypertension (maximum 140/87 mmHg). Blood smears showed pronounced basophilic stippling of erythrocytes (Figure 1A). Blood lead levels (BLLs) were determined and markedly elevated at 123 µg/dl (upper limit of normal 3.5 µg/dl).

**Figure 2:**
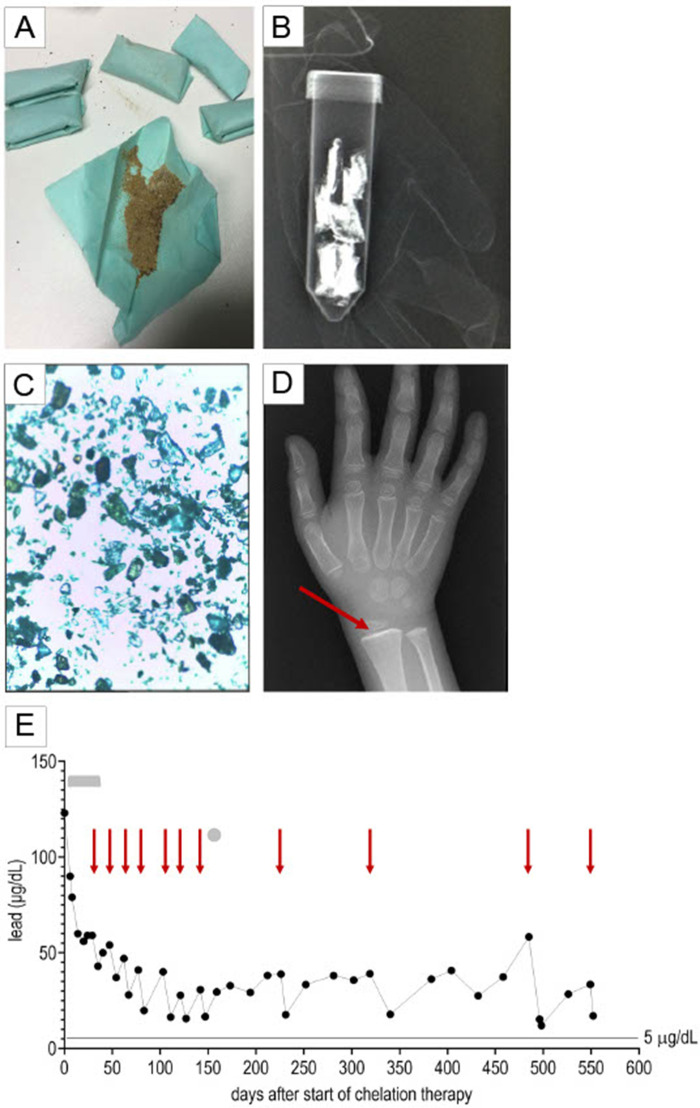
Lead poisoning. The patient ingested an ayurvedic herbal medicine termed *Indian spices*. **(A)** The *Indian spices* were provided as a brown powder wrapped in filter paper. **(B)** The packets were found to be radio-opaque. **(C)** The powder contained crystalline elements. **(D)** An x-ray of the wrist demonstrated a lead line in the distal radius metaphysis (marked by a red arrow). **(E)** Blood lead levels slowly decreased after initiation of chelation therapy with intravenous 2,3-dimercapto-1-propanesulfonic acid (DMPS, denoted by a grey bar) and subsequent intravenous CaNa_2_EDTA administration in 5-day cycles (start of CaNa_2_EDTA cycles marked by red arrows). One dose of oral DMPS was administered (marked by a grey circle). Intravenous DMPS was discontinued due to development of erythema multiforme; oral DMPS was discontinued after a single dose because of an allergic reaction. The gray bar indicates the period of intravenous DMPS therapy, the gray circle indicates the single dose of oral DMPS, and the red arrows indicate the start of CaNa_2_EDTA cycles.

Upon further questioning, the parents reported seasonal use of an ayurvedic herbal medicine (termed *Indian spices*) for treatment of asthma over the course of three winter seasons (approximately 4–5 months per season). The herbal medicine—a brown powder wrapped in filter paper (Figure 2A)—was imported by a non-licensed healer from India and distributed at a German yoga school. The packets were radiopaque (Figure 2B) consistent with a lead content of at least 2.2% (sample 1: 21,500 mg/kg; sample 2: 13,75 mg/kg sample 3: 6,16 mg/kg) and a mercury content of 0,73% (sample 1: 7,300 mg/kg; sample 2: 4,700 mg/kg; sample 3: 6,500 mg/kg). Significant discrepancies in lead content between samples correlated with macroscopic (color, consistency) and microscopic differences (Figure 2C). The lead and mercury concentrations reported above reflect the amount of lead and mercury in powder that was dissolved in nitric acid. The actual lead and mercury content could be higher, because the powder could not be dissolved completely. The patient's blood and urine mercury levels were within the reference range. An abdominal x-ray did not demonstrate lead lining of the patient's bowel walls, but x-rays of the wrist demonstrated a lead line in the metaphysis of the distal radius consistent with hypercalcification in the zone of provisional calcification, due to inhibition of osteoclasts by bony accumulation of lead (Figure 2D). The patient's parents and sister had blood and urine lead/ mercury levels within the reference range. They had not ingested the ayurvedic medicine. Legal authorities were informed of the case.

Chelation therapy with intravenous 2,3-dimercapto-1-propanesulfonic acid (DMPS, 5 mg/kg/doses/8 h) resulted in rapid clinical improvement, but caused mild hepatotoxicity (maximum Glutamate Oxaloacetate Transaminase 257 U/L, Glutamate Pyruvate Transaminase 272 U/L) and development of erythema multiforme after one month of treatment. Symptoms resolved after discontinuation of DMPS and start of intravenous calcium disodium EDTA (CaNa_2_ EDTA, 1,500 mg/m^2^/day continuous infusion for 5 days per cycle). The patient remained hospitalized for the entire DMPS treatment and the first cyle of CaNa_2_ EDTA (35 days). Subsequently, he was readmitted for CaNa_2_ EDTA infusions. After seven CaNa_2_ EDTA cycles, oral DMPS became available in Germany. Yet, after an allergic reaction to the first oral DMPS dose, treatment with CaNa_2_ EDTA had to be resumed. The precise substance in the DMPS preparation, which caused the allergic reaction, is not known. Treatment with oral dimercaptosuccinic acid (DMSA)—which is structurally different from DMPS—was refused by the family. Declining BLLs were observed after each chelation cycle, but BLLs rebounded in between, suggesting redistribution from skeletal lead stores. Eighteen months (552 days) after starting chelation, BLLs were recorded at 33.4 µg/dl prior and 17.0 µg/dl immediately after completing the 11th CaNa_2_ EDTA cycle (Table 1).

**Table 1:** Blood lead levels (BLLs) over time.

Day	BLL (µg/L)	Comment
0	1,230	Intravenous DMPS started
6	900	
8	790	
14	600	
20	560	
24	590	Erythema multiforme, intravenous DMPS stopped
29	590	CaNa_2_ EDTA cycle #1
35	430	
40	500	
47	540	CaNa_2_EDTA cycle #2
54	370	
62	470	CaNa_2_EDTA cycle #3
67	280	
77	410	CaNa_2_EDTA cycle #4
83	198	
103	400	CaNa_2_EDTA cycle #5
111	164	
121	278	CaNa_2_EDTA cycle #6
127	156	
142	307	CaNa_2_EDTA cycle #7
147	166	
159	295	Oral DMPS ×1 dose followed by allergic reaction
173	328	
194	292	
212	382	
226	388	CaNa_2_EDTA cycle #8
231	176	
252	333	
281	381	
302	357	
319	390	CaNa_2_EDTA cycle #9
340	178	
383	362	
404	407	
432	275	
458	373	
485	583	CaNa_2_EDTA cycle #10
496	152	
498	120	
526	284	
549	334	CaNa_2_EDTA cycle #11
552	170	

Initial neurocognitive assessment was performed approximately 30 days after the initiation of chelation therapy and showed a non-verbal IQ of 112 (Snijders-Oomen test; range: 104–119). Follow-up after 1,5 years revealed emotional lability and delays in gross motor coordination. The patient remains under close surveillance to monitor for potential long-term effects.

## Discussion

Ayurveda—one of the oldest traditional systems of medicine originating in India—has gained traction in Western countries as a consequence of a broader trend towards complementary and alternative medicine (CAM) and due to immigration of people who continue to use traditional remedies widely used in their countries of origin. While CAM is subject to strict controls regarding quality, manufacturing, and distribution, Ayurvedic products often lack such oversight and may be purchased without medical consultation via ethnic markets, health food stores, Ayurveda practitioners, and online platforms. Their perception as “natural” and “safe” contributes to the risk of exposure to potentially harmful substances. In the Rasa Shastra tradition, heavy metals such as lead, arsenic, mercury, and cadmium—known as bhasmas—are believed to have therapeutic benefits when “purified” (suddha) through ancient detoxification techniques (1). Consequently, ayurvedic remedies may contain alarming levels of heavy metals. Recent studies have shown that the lead, mercury, or arsenic content of approximately 20% of ayurvedic medicines available online exceeds the safety limits set by the World Health Organization (WHO). Possible adverse effects include neurotoxicity, nephrotoxicity, hematological abnormalities, and impaired child development (5). Pregnant women and children are at particular high risk (1).

Beyond ayurvedic supplements, alternative causes of lead poisoning include lead-based wall paint, contaminated soil or dust, lead-laden water pipes, industrial emissions, and other folk remedies or cosmetics (4). Clinically, lead poisoning may manifest with encephalopathy, arterial hypertension, abdominal pain, constipation, and anorexia. Laboratory abnormalities—especially with higher BLLs—include anemia and basophilic stippling. Liver and kidney dysfunction may occur with elevated liver enzymes, hyperbilirubinemia, elevated creatinine levels, hyponatremia, and proteinuria (6). In the case reported here, basophilic stippling on blood smears and classic symptoms including abdominal pain, arterial hypertension and early signs of encephalopathy led the way to the diagnosis. Still, as environmental lead exposures have not been given much public attention in Germany, the diagnosis was unexpected and highlights the need for greater awareness of the signs and symptoms of lead poisoning.

Approximately 10%–15% of ingested inorganic lead (commonly found in environmental sources such as water and soil) and up to 30% of ingested organic lead are absorbed from the gastrointestinal tract. Other factors influencing absorption include age and nutritional status. Gastrointestinal absorption of lead may reach up to 50% in children (9). At the cellular level, lead disrupts various biological functions, promoting oxidative damage to DNA and membranes, impairing transcription, and possibly inhibiting vitamin D synthesis, which is essential for maintaining cell membrane integrity (6). Lead is a potent neurotoxin and accumulates in the brain, kidneys, liver, and bones. As children's blood-brain barrier is less effective at preventing lead from entering the brain (8), lead is especially harmful in children. Current research shows that lead exposure—even at low BLLs—disrupts neurodevelopmental processes, including synapse formation, neurotransmitter function, and myelination (10).

The half-life of lead is approximately 30 days in blood and approximately 15 years in cortical bones (e.g., the femur) (7). Skeletal lead stores may release lead into the bloodstream and thereby contribute to elevated BLLs in the absence of ongoing external exposure, as evidenced by persistently elevated BLLs in the patient reported here. This phenomenon substantially complicates efforts to manage and mitigate the risks associated with lead poisoning (6). Historically, lead-induced damage has been considered irreversible, but emerging evidence indicates that some neurodevelopmental deficits may be partially improved with appropriate interventions (10). Sustained exposure to substantially elevated BLLs in the patient reported here raises concern and will require long-term monitoring.

## Conclusion

This report highlights the risk of severe lead poisoning secondary to ayurvedic supplements. Healthcare providers should remain vigilant and inquire about CAM use, especially in vulnerable populations such as children. Better strategies to monitor the quality and composition of alternative remedies and to educate families about possible heavy metal contamination could mitigate health risks.

## Data Availability

The original contributions presented in the study are included in the article/Supplementary Material, further inquiries can be directed to the corresponding author.
